# Epigenetic mechanisms and clinical translation in ovarian cancer: from molecular pathways to precision therapy

**DOI:** 10.1042/BSR20250409

**Published:** 2026-08-25

**Authors:** Khaldoon Alsamman

**Affiliations:** Department of Clinical Laboratory Sciences, College of Applied Medical Sciences, Imam Abdulrahman Bin Faisal University, Dammam, Saudi Arabia

**Keywords:** Chemoresistance, Clinical translation, DNA methylation, Epigenetic modifications, Ovarian cancer

## Abstract

Ovarian cancer remains one of the deadliest gynecological malignancies, mainly due to its late diagnosis, biological heterogeneity, and frequent development of chemoresistance. Recent evidence indicates that epigenetic dysregulation including aberrant DNA methylation, histone modifications, chromatin remodeling, and non-coding RNA networks plays an important role in the initiation, progression, and therapeutic response of ovarian cancer. Unlike genetic alterations, epigenetic modifications are reversible, making them attractive targets for drug development and precision oncology. In this review, we provide a detailed overview of the epigenetic mechanisms that control ovarian cancer biology, with specific focus on their role in drug resistance and disease recurrence. We discuss the role of epigenetic plasticity in enabling transition of tumor cells into drug tolerant, stem like phenotypes, leading to minimal residual disease and eventual relapse without permanent genetic changes. Attention is given to the influence of DNA methyltransferases, histone deacetylases, enhancer of zeste homolog 2, and chromatin remodeling complexes in the maintenance of adaptive resistance programs. We also discuss the evolving paradigm of epigenetic regulation, immune modulation and viral mimicry and provide the rationale for combining epigenetic therapies with immunotherapy and poly(ADP-ribose) polymerase inhibition. The article also reviews the results of recent clinical trials and evaluate the most recent advances in biomarker development, such as circulating DNA methylation signatures, microRNAs, and artificial intelligence-based liquid biopsy platforms. Finally, we discussed the potential implications of applying epigenetic profiling in the clinic for improved patient stratification and the potential for real-time monitoring of chemoresistance. These progresses make epigenetic regulation a promising strategy to overcome chemoresistance and improve personalized medicine of ovarian cancer.

## Introduction

Ovarian cancer remains one of the most lethal gynecologic malignancies worldwide, with epithelial ovarian cancer (EOC) constituting the predominant histological subtype [1]. Most patients experience recurrence of their platinum-based chemotherapy resistant disease despite an initial response, posing a major clinical challenge and affecting long-term survival rates. Emerging evidence suggests that epigenetic dysregulation plays a critical role in promoting ovarian cancer progression and therapeutic resistance through mechanisms including DNA methylation, histone modifications, and non-coding RNAs (ncRNAs) [2,3].

Epigenetic modifications refer to heritable, yet reversible, changes in gene expression that do not involve alterations in the underlying DNA sequence. These modifications include DNA methylation, histone tail modifications (e.g., acetylation and methylation), and regulation by ncRNAs such as microRNAs (miRNAs), long non-coding RNAs (lncRNAs), and circular RNAs (circRNAs). In cancer, these mechanisms are frequently hijacked to suppress tumor suppressor genes, activate oncogenes, or alter the chromatin landscape, contributing to uncontrolled growth, immune evasion, metastasis, and drug resistance [4,5].

Growing evidence indicates a significant role for epigenetic alterations in the development of chemoresistance in EOC. Decreased sensitivity to chemotherapeutic agents, particularly platinum-based drugs, has been linked to DNA hypermethylation of repair genes, such as *BRCA1* and *MLH1* [6,7]. Similarly, alterations in the pattern of histone deacetylation and methylation, as well as the role of enhancer of zeste homolog 2 (EZH2) and the accumulation of repressive H3K27me3 marks have been implicated in the maintenance of a chemo-resistant phenotype [8,9]. Dysregulation of specific miRNAs, including the miR-200 family, *miR-21* and *let-7*, has been associated with aberrant apoptotic signaling, induction of epithelial–mesenchymal transition (EMT) and increased expression of multidrug resistance proteins [10,11].

This review discusses epigenetic mechanisms that contribute to chemotherapy resistance and progression of ovarian cancer, and their translational and clinical implications. To give a full picture, we also include results from narrative reviews, original research, and meta-analyses obtained by a systematic literature review. The review is organized in five themes: (i) the epigenetic landscape of ovarian cancer; (ii) the role of DNA methylation, histone modifications, and ncRNAs in modulating therapeutic responses and tumor evolution; (iii) the development and clinical validation of epigenetic biomarkers for diagnosis, prognosis, and treatment monitoring; (iv) therapeutic strategies targeting epigenetic regulators including combination approaches with poly(ADP-ribose) polymerase (PARP) and immune-checkpoint inhibitors; and (v) emerging translational and clinical applications including liquid biopsy technologies, AI-driven analytics, and personalized epigenetic therapies. This review aims to shed light on the intricate relationship between epigenetic regulation and chemoresistance in ovarian cancer, opening doors for biomarker-guided interventions and novel combination therapies to enhance clinical outcomes and foster precision oncology.

## Core epigenetic mechanisms in ovarian cancer progression and chemoresistance

Epigenetic regulation in ovarian cancer involves multiple levels of gene expression regulation, including DNA methylation, histone modifications, and interactions with ncRNAs. These regulatory mechanisms, which do not modify the DNA sequence to influence gene activity, are critical in defining tumor behavior, progression, and therapeutic responses [5,12]. Besides tumorigenesis, epigenetic modifications are being increasingly acknowledged as contributors to acquired drug resistance, via the modulation of pathways involved in DNA repair, apoptosis, drug efflux, EMT, and survival mechanisms. These alterations allow tumor cells to adapt to therapeutic stress and evade treatment-induced cytotoxicity [1,4]

### DNA methylation in tumor progression and drug response

DNA methylation, particularly of CpG islands in gene promoters, is an important regulator of gene silencing. Consistent reports of hyper-methylation of tumor suppressor genes and genes involved in DNA repair pathways including *BRCA1, MLH1, MGMT*, and *RAD51C* [7] characterize ovarian cancer [1,7]. Such epimutations lead to genomic instability and contribute to oncogenesis. *BRCA1* promoter hypermethylation has been described in several studies and is associated with defective homologous recombination repair, increased sensitivity to DNA damaging agents, and resistance upon change of reversion or methylation status [13,14]. During disease progression, methylation of genes related to EMT and tumor suppression (*SFRP5, HSulf-1*, and *RASSF1A*) may promote EMT, invasion, and dissemination. In contrast, hypomethylation of proto-oncogenes and repetitive DNA sequences can lead to chromosomal instability, and subsequently enhance metastatic potential [7,15,16].

Many studies have reported hypermethylation of tumor suppressor genes, which leads to increased proliferation and invasion. Methylation of *BRCA1*, *MLH1*, and other DNA repair genes results in genomic instability and is associated with tumor aggressiveness and metastasis [5,7]. Additionally, hypomethylation of proto-oncogenes and repetitive DNA sequences has been associated with chromosomal instability and an increased metastatic potential [13]. Genes involved in EMT, such as *SFRP5*, *HSulf-1*, and *RASSF1A*, are differentially methylated, supporting the transition from epithelial-to-mesenchymal phenotypes, promoting detachment, invasion, and dissemination of cancer cells [7,14].

Platinum resistance in ovarian cancer is significantly associated with hypermethylation of DNA repair genes such as *BRCA1, MLH1, RAD51C*, and *MGMT* [5,15]. Methylation-mediated silencing of *BRCA1* disrupts homologous recombination repair (HRR), which may initially make ovarian tumors more sensitive to platinum-based chemotherapy. However, during disease progression or relapse, loss of *BRCA1* promoter methylation or reactivation of *BRCA1* expression can restore DNA repair capacity, thereby contributing to acquired platinum resistance [16]. *MLH1* hypermethylation has also been associated with reduced mismatch repair capacity and plays a role in cisplatin resistance [7]. On the contrary, hypomethylation of genes such as *MBNL2* was shown to inhibit apoptosis and increase cisplatin resistance [17,18]. These findings demonstrate the dual nature of methylation alterations and their complex role in influencing drug response.

Ovarian cancer pathogenesis involves complex DNA methylation that may lead to the inappropriate activation of oncogenes and the silencing of tumor suppressor genes. Nevertheless, genetic *BRCA1/2* mutations, *BRCA1* promoter methylation and functional homologous recombination deficiency (HRD) should not be considered equivalent. *BRCA1/2* genetic mutations are sequence changes that may be germline or somatic. *BRCA1* promoter methylation is an epigenetic mechanism of transcriptional silencing that might be reversible and exhibit heterogeneity within tumors. Functional HRD is a phenotype associated with downstream DNA repair, which can be caused by *BRCA* mutation, *BRCA1* promoter methylation, *RAD51C* methylation, or other Homologous recombination repair (HRR) defects. This distinction is clinically relevant, as epigenetic silencing may initially confer sensitivity to platinum-based chemotherapy and PARP inhibitors while demethylation, loss of methylation or restoration of DNA repair capacity may lead to acquired resistance upon relapse [5,13–15].

It is important to distinguish genetic *BRCA1/2* mutation from *BRCA1* methylation and functional HRD as these terms are not interchangeable. Genetic changes in *BRCA1/2* are sequence-level defects that can be germline or somatic, whereas *BRCA1* promoter methylation is an epigenetic mechanism of transcriptional silencing that can be reversible and heterogeneous within a tumor. The downstream DNA repair phenotype is defined by functional HRD and could be caused by *BRCA* mutations, *BRCA1* promoter methylation, *RAD51C* methylation or other defects impacting homologous recombination repair. This difference has clinical implications as epigenetic silencing may increase sensitivity to platinum and PARP inhibitors initially, while demethylation, loss of methylation, or restoration of DNA repair capacity may lead to acquired resistance upon relapse [5,13,15,16].

### Histone modifications, chromatin remodeling, and resistance

Histone modifications play a central role in regulating chromatin compaction and transcriptional accessibility through processes such as acetylation and methylation of histone tails. In ovarian cancer, epigenetic regulation is largely mediated by histone-modifying enzymes, including histone deacetylases (HDACs) and histone methyltransferases such as EZH2. Aberrant HDAC activity promotes global hypoacetylation and repression of apoptosis-related genes [9], whereas EZH2-mediated trimethylation of histone H3 lysine 27 (H3K27me3) contributes to gene silencing in drug-resistant cells [9,19]. Emerging evidence has further highlighted the importance of additional histone marks, including H3K4me3 and H3K79me, as well as post-translational modifications such as phosphorylation and ubiquitination, in regulating gene expression programs associated with cancer stemness, EMT, immune evasion, and therapeutic resistance [3,8]. These dynamic histone modifications act as regulatory hubs, integrating environmental and intracellular signals to modulate oncogenic programs.

Epigenetic plasticity allows ovarian cancer cells to dynamically reprogram transcriptional states without the need to acquire additional genetic alterations. Such plasticity is increasingly acknowledged as a key driver of disease progression, metastatic potential, and therapy resistance. Changes in chromatin regulatory complexes, particularly members of the SWI/SNF family, affect the accessibility and fidelity of enhancers, and permit tumor cells to survive oncogenic stress and environmental challenges. Thus, in *ARID1A*-mutated ovarian cancer, the loss of chromatin remodeling function imparts a dependency on compensatory epigenetic regulators, rather than stable genetic drivers, highlighting the importance of chromatin state as a key determinant of tumor behavior [20].

Mechanistically, *ARID1A* deficiency induces aberrant patterns of histone acetylation and enhancer dysregulation that promote transcriptional programs associated with survival and lineage plasticity. Bitler et al. demonstrated that *ARID1A*-mutant ovarian cancer cells depend on histone deacetylase 6 (HDAC6) activity for the maintenance of oncogenic signaling and cellular viability [20]. This finding highlights a context-specific chromatin dependency driven by epigenetic imbalance rather than mutational burden and underscores the role of epigenetic mechanisms in sustaining tumor growth and adaptability.

Histone-modifying enzymes are key contributors to ovarian cancer progression and treatment resistance. EZH2 promotes H3K27me3-mediated transcriptional repression and has been implicated in the induction of EMT and silencing of tumor suppressor pathways [9,21]. In addition, EZH2 overexpression has been associated with platinum resistance and poor clinical outcomes in ovarian cancer patients [19]. Increased histone deacetylation mediated by HDACs results in chromatin condensation and transcriptional repression of genes involved in metastasis suppression and invasion control [8]. In resistant tumors, EZH2-mediated H3K27me3 may silence tumor suppressor and pro-apoptotic genes [9,19], whereas HDAC-induced chromatin compaction may impair apoptotic signaling and DNA damage response pathways [9]. Together, these observations provide a strong rationale for targeting EZH2 and HDACs as therapeutic strategies to overcome treatment resistance [9,19,21]

Further histone modifications increase the aggressiveness of ovarian cancer and its resistance to therapy. Histone modifications such as H3K4me3 and H3K79me are associated with active transcription of genes that promote cancer stem cell properties and induce resistance to apoptosis [3]. Furthermore, H3K4me3, H3K79me, and certain phosphorylation signatures have been linked to the regulation of DNA repair pathways and resistance to PARP inhibitors [3]. These findings reveal a dual role of histone modifications in repressing tumor suppressor pathways and activating oncogenic networks to promote disease progression and treatment failure. Therefore, therapeutic approaches targeting histone-modifying enzymes, such as HDAC inhibitors [2,21] and EZH2 inhibitors [9,21], have emerged as promising approaches to overcome epigenetically induced drug resistance.

### Non-coding RNAs in EMT, metastasis, and chemoresistance

ncRNAs, including miRNAs, lncRNAs, and circRNAs, are important epigenetic regulators of gene expression and play critical roles in ovarian cancer progression and therapeutic resistance. Dysregulation of several miRNAs, including the miR-200 family, miR-21, let-7, miR-214, and miR-142-5p, has been associated with cell survival, proliferation, metastasis, EMT, apoptosis regulation, and drug resistance [2,10,14,22,23].

The miR-200 family is a key regulator of EMT through suppression of the transcription factors ZEB1 and ZEB2 [2,22]. Down-regulation of miR-200 family members promotes mesenchymal transformation, resulting in increased invasiveness, metastatic potential, and reduced drug sensitivity [2,22]. In contrast, up-regulation of miR-21 has been associated with inhibition of apoptosis and activation of oncogenic signaling pathways that support tumor cell survival [11]. Additional miRNAs, including let-7, miR-214, and miR-142-5p, have been implicated in the regulation of apoptosis, EMT, DNA repair, and drug efflux mechanisms in chemotherapy-resistant ovarian cancer [10,14,23]. Notably, reduced expression of miR-142-5p leads to overexpression of anti-apoptotic targets such as *BCL2* and *MCL1*, thereby promoting platinum-based therapy resistance [23]

Beyond miRNAs, lncRNAs and circRNAs contribute to ovarian cancer pathogenesis through diverse regulatory mechanisms. lncRNAs frequently function as competing endogenous RNAs (ceRNAs), sequestering tumor-suppressive miRNAs and indirectly enhancing oncogene expression [24] Similarly, circRNAs can act as miRNA sponges to regulate gene networks involved in proliferation, invasion, and metastatic progression. In addition, both lncRNAs and circRNAs may influence chromatin structure and gene expression through the recruitment of chromatin-modifying complexes and chromatin remodelers [17,18]. The interactions between ncRNAs and their epigenetic targets form complex regulatory networks that contribute to ovarian cancer progression and chemoresistance (Table 1).

**Table 1:** Summary of key epigenetic modifications in ovarian cancer

Epigenetic mechanism	Specific alteration	Key genes/targets	Functional outcome	Associated phenotype
DNA methylation	Hypermethylation	*BRCA1, MLH1, RAD51C*	Silencing of DNA repair/tumor suppressors	Chemoresistance and progression
Histone modification	H3K27me3 via EZH2	Global repression	Chromatin compaction and gene silencing	EMT and drug resistance
Non-coding RNAs	miR-21 up-regulation	*PTEN, BCL2*	Apoptosis inhibition	Platinum-based therapy resistance
Non-coding RNAs	miR-200 down-regulation	*ZEB1, ZEB2*	EMT activation	Metastasis and resistance

Dysregulation of miRNAs such as miR-200 family, let-7, miR-21, and miR-214 are tightly associated with EMT and metastasis. In normal conditions, the miR-200 family suppresses EMT transcription factors ZEB1 and ZEB2. The down-regulation of the miR-200 family promotes a mesenchymal transformation that increases invasiveness [2,25]. Similarly, up-regulation of miR-21 has been correlated with inhibition of apoptosis and activation of oncogenic pathways. Another layer of complexity is lncRNA–miRNA interactions. lncRNAs act as ceRNAs to sponge tumor-suppressive miRNAs and indirectly promote oncogene expression. circRNAs have been shown to act as miRNA sponges to modulate gene networks associated with invasion and proliferation [24,26].

Chemotherapy resistance, particularly to platinum-based agents such as cisplatin and carboplatin, remains a major challenge in the management of ovarian cancer. Epigenetic alterations contribute to the development of acquired resistance through reversible and dynamic mechanisms that regulate the expression of genes involved in drug efflux, DNA repair, apoptosis, and cellular survival [1,4]. These alterations include DNA methylation, histone modifications, and dysregulation of ncRNAs, which collectively influence tumor cell behavior and therapeutic response as summarized in Table 2.

**Table 2:** Epigenetic modifications associated with chemotherapy resistance

Mechanism	Epigenetic marker	Drug resistance type	Mechanism of resistance	References
DNA methylation	BRCA1, MLH1	Platinum-based therapies	Impaired DNA repair	[5,7,15]
Histone modification	H3K27me3 (EZH2)	Platinum-based therapies	Transcriptional repression	[9,19]
miRNA	miR-21, miR-200, let-7	Cisplatin and others	EMT and apoptosis evasion	[10,11,14]

Importantly, these epigenetic mechanisms do not operate independently. DNA methylation regulates the expression of multiple miRNAs, whereas miRNAs can directly target DNA methyltransferases (DNMTs) and histone-modifying enzymes, creating feedback loops that reinforce resistant phenotypes [12]. Furthermore, lncRNAs and circRNAs contribute to chemoresistance by modulating miRNA availability and recruiting chromatin-remodeling complexes [24,26]. This extensive cross-talk highlights the integrated nature of epigenetic regulation in ovarian cancer.

Among ncRNAs, miRNAs are particularly important mediators of chemoresistance. Several miRNAs, including the miR-200 family, miR-21, let-7, miR-214, and miR-142-5p, regulate pathways involved in apoptosis, EMT, DNA repair, and drug transport [4,10]. Up-regulation of miR-21 suppresses pro-apoptotic genes and enhances cellular survival in response to cisplatin treatment [11]. Conversely, down-regulation of miR-142-5p results in increased expression of anti-apoptotic proteins such as BCL2 and MCL1, thereby promoting resistance to therapy [23]. The miR-200 family is frequently down-regulated in resistant tumors and plays a critical role in regulating EMT and drug sensitivity, making it a promising therapeutic target for overcoming chemoresistance [2,22].

### Epigenetic regulation of tumor cell plasticity, minimal residual disease, and intratumoral heterogeneity

Epigenetic plasticity enables ovarian cancer cells to dynamically reprogram transcriptional states without acquiring new genetic alterations and is increasingly recognized as a key driver of disease progression, metastatic dissemination, and therapeutic resistance. Disruption of chromatin-regulatory complexes, particularly members of the SWI/SNF family, alters enhancer accessibility and transcriptional fidelity, thereby facilitating adaptation to oncogenic stress and microenvironmental pressures. In *ARID1A*-mutated ovarian cancer, loss of chromatin-remodeling activity creates a dependency on compensatory epigenetic regulators rather than stable genetic drivers, highlighting the critical role of chromatin state in determining tumor behavior [20].

Epigenetic plasticity also contributes to the emergence of drug-tolerant persister cells characterized by altered chromatin accessibility, attenuated apoptotic signaling, and enhanced stress resistance. DNA methylation plays a central role in this process, as partial or heterogeneous methylation of DNA repair and apoptosis-related genes can reversibly suppress chemosensitivity pathways. Variable methylation states of *BRCA1* and *MLH1* may therefore permit residual tumor cells to survive chemotherapy and subsequently repopulate the tumor following treatment cessation, providing a molecular basis for disease relapse after an initial therapeutic response [5,16]. Histone modifications further reinforce adaptive resistance states by reshaping enhancer and promoter landscapes. Repressive histone marks, including EZH2-mediated H3K27me3, silence differentiation-associated and pro-apoptotic genes, whereas activating marks such as H3K4me3 and H3K79me promote transcriptional programs associated with survival and stemness [3,9,19].

Loss of *ARID1A* further amplifies tumor cell plasticity through disruption of lineage fidelity and enhancer regulation. Bitler et al. demonstrated that *ARID1A*-deficient ovarian cancer cells depend on HDAC6 activity to maintain adaptive transcriptional programs that support survival under therapeutic stress [20] This context-specific chromatin dependency suggests that minimal residual disease (MRD) may be sustained by epigenetic imbalance rather than ongoing genetic evolution.

Non-coding RNAs also contribute to tumor cell plasticity and persistence by fine-tuning gene expression networks involved in EMT, apoptosis, and DNA damage responses. As previously recounted, down-regulation of the miR-200 family promotes EMT and acquisition of stem-like features, whereas up-regulation of miR-21 suppresses apoptotic signaling and enhances drug tolerance [10,22]. lncRNAs and circRNAs further strengthen these adaptive programs by functioning as ceRNAs and by recruiting chromatin-modifying complexes to resistance-associated loci [24,26]. Together, these regulatory interactions enable ovarian cancer cells to transition dynamically between drug-sensitive and drug-resistant phenotypic states.

Clinically, epigenetically regulated MRD has important implications for disease monitoring and treatment strategies. Residual tumor cells maintained in chromatin-based plasticity states may evade detection by conventional imaging modalities yet remain identifiable through liquid biopsy approaches that capture dynamic epigenetic alterations. Monitoring circulating cell-free DNA (cfDNA) methylation patterns and circulating miRNA profiles may therefore provide early indicators of emerging resistance and facilitate timely therapeutic intervention [27,28]. Moreover, the reversible nature of these epigenetic states provides a strong rationale for incorporating epigenetic therapies as priming or maintenance strategies aimed at eliminating MRD and preventing disease recurrence.

Intratumoral heterogeneity in ovarian cancer is shaped by both genetic and epigenomic variation, with spatially and temporally distinct chromatin states influencing therapeutic response. Recent single-cell chromatin accessibility studies of high-grade serous ovarian cancer metastases have demonstrated that residual tumor cells surviving chemotherapy undergo extensive regulatory remodeling, including enrichment of accessible chromatin regions associated with stress-response pathways and resistance-related transcription factor programs [29,30]. These findings support a model in which chemotherapy not only selects for pre-existing resistant clones but also remodels the epigenomic landscape of surviving cells, promoting the expansion of adaptive regulatory states after treatment [29,30].

**Figure 1:**
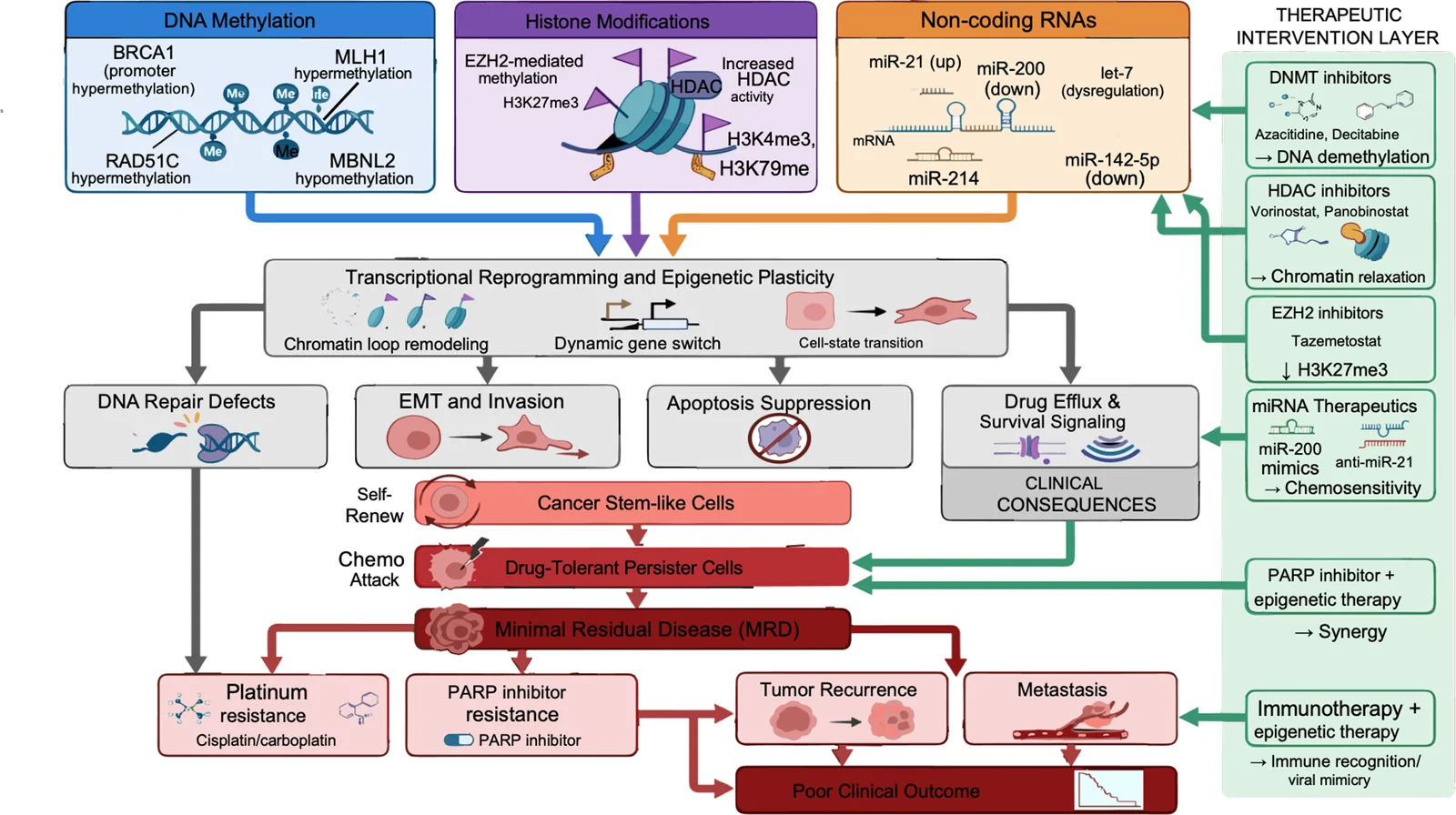
Epigenetic drivers of ovarian cancer progression, tumor plasticity, and chemotherapy resistance Epigenetic drivers of ovarian cancer progression, tumor plasticity, and chemotherapy resistance. Aberrant DNA methylation, histone modifications, and dysregulated ncRNAs cooperatively remodel transcriptional programs that govern DNA repair, apoptosis, EMT, stemness, and survival signaling. These alterations promote epigenetic plasticity and adaptive cell-state transitions, leading to the emergence of cancer stem-like cells and drug-tolerant persister populations. Persistence of these adaptive states contributes to MRD, intratumoral heterogeneity, platinum resistance, PARP inhibitor resistance, tumor recurrence, and metastatic progression. The therapeutic intervention layer highlights major epigenetic strategies, including DNMT, HDAC, and EZH2 inhibitors, as well as miRNA-based therapies and combination approaches with PARP inhibitors or immunotherapy, which may reverse resistance-associated chromatin states and restore treatment sensitivity.

Consistent with this concept, single-nucleus multiomic profiling studies suggest that chemotherapy-resistant phenotypes may be epigenetically primed before treatment initiation. Longitudinal analyses of high-grade serous ovarian cancer samples have shown that chemotherapy is associated with depletion of proliferative and interferon-responsive cellular states and expansion of Tumor necrosis factor-α (TNFα) and EMT-associated programs [29,30]. Furthermore, H3K4me1-marked enhancer landscapes present before treatment exposure appear to predispose subsets of tumor cells to resistant phenotypic states. These observations establish a mechanistic link between baseline non-genetic tumor composition, adaptive transcriptional remodeling, and clinical outcome. Consequently, precision epigenetic therapies should target not only static biomarkers such as *BRCA1* methylation and *EZH2* expression but also the dynamic chromatin states that evolve under therapeutic pressure [29,30]. The interrelationship between these epigenetic mechanisms and their contribution to disease evolution is summarized in (Figure 1).

## Diagnostic and prognostic biomarkers

The search for reliable biomarkers in ovarian cancer has intensified to improve early diagnosis, evaluate therapy response, and predict clinical outcomes. Epigenetic changes are promising biomarker candidates because they occur early, are dynamically regulated and detectable in biological fluids (Table 3). DNA methylation profiles, histone modification patterns, and ncRNAs, especially circulating miRNAs, have emerged as major candidates for diagnostic and prognostic purposes [1,4].

**Table 3:** Potential epigenetic biomarkers in ovarian cancer

Biomarker type	Specific marker	Sample type	Diagnostic/Prognostic utility	Clinical relevance
DNA methylation	BRCA1, MBNL2	Tumor tissue and blood	Predictive of platinum-based therapy response	Stratification
miRNA	miR-21, miR-142-5p	Serum/plasma	Predictive and prognostic	Liquid biopsy candidate
Histone modification	EZH2 expression, H3K27me3	Tumor tissue	Prognostic marker	Therapy stratification

### DNA methylation biomarkers

Hypermethylation of genes like *BRCA1*, *MLH1*, *MGMT*, and *RAD51C* has been suggested as a predictive biomarker for platinum-based therapy response [5,15]. *BRCA1* methylation is frequently linked to initial platinum sensitivity, and loss of methylation at relapse may indicate acquired resistance [16]. *MBNL2* hypomethylation was identified as a potential prognostic biomarker for cisplatin resistance and poor outcomes [17,18]. Additional methylated loci, such as *SFRP5*, *RASSF1A*, and *HSulf-1*, were associated with early tumorigenesis and may facilitate early detection [7]. Methylation panels are particularly useful in liquid biopsy applications for non-invasive screening and monitoring of disease.

### MicroRNAs as circulating biomarkers

One of the most promising epigenetic biomarkers in ovarian cancer is circulating miRNAs. miRNAs, such as miR-21, the miR-200 family, let-7, miR-142-5p, and miR-214, have been reported to be differentially expressed in plasma/serum samples of patients in several studies [22,31,32]. These miRNAs have demonstrated the ability to distinguish chemo-resistant from chemo-sensitive tumors, and to predict patient survival. For example, platinum-based therapies-resistant cases frequently demonstrate increased circulating miR-21 and decreased miR-200 levels [10]. Likewise, miR-142-5p has been reported to regulate apoptosis-related genes and its down-regulation is related to unfavorable prognosis [23]. Therefore, circulating miRNAs could serve as a non-invasive tool for assessing treatment response and relapse.

### Histone modifications and other epigenetic markers

Histone modifications have been less studied as direct biomarkers, but their prognostic relevance has been examined, particularly H3K27me3, which is regulated by *EZH2*. EZH2 and related methylation markers have been associated with poor prognosis and resistance to therapy [9,21]. Some studies suggest that they may be used in the future to stratify patients for epigenetic therapy trials. Furthermore, lncRNAs and circRNAs are emerging as potential biomarkers owing to their stability in biofluids and their participation in resistance pathways. However, their diagnostic performance still needs to be validated in large clinical cohorts [24,26].

## Therapeutic targeting of epigenetic modifiers

The reversibility of epigenetic modifications offers a significant potential for therapeutic intervention in ovarian cancer, especially for overcoming chemotherapy resistance. Targeting DNMTs, histone-modifying enzymes, and non-coding RNA networks represents a promising strategy to restore chemosensitivity, reactivate silenced tumor suppressors, and regulate critical oncogenic pathways. Several epigenetic agents are under preclinical or clinical evaluation as single agents or as part of combination therapy with standard chemotherapy (Table 4) [3,5].

**Table 4:** Therapeutic strategies targeting epigenetic modifiers

Therapeutic class	Agent/Approach	Epigenetic target	Mechanism	Stage of development
DNMT inhibitor	Decitabine and azacitidine	DNA methylation	Reactivate silenced genes	Preclinical/clinical
HDAC inhibitor	Vorinostat and panobinostat	Histone deacetylation	Chromatin relaxation	Clinical trials
EZH2 inhibitor	Tazemetostat	H3K27me3	Inhibits repressive histone methylation	Investigational
miRNA modulation	miR-200 mimics and anti-miR-21	Post-transcriptional regulators	Apoptosis/EMT modulation	Preclinical

In addition to the tumor intrinsic transcriptional regulation, epigenetic mechanisms also play an important role in immune recognition in ovarian cancer. Epigenetic regulation also involves the non-malignant cellular component of the ovarian tumor microenvironment. Signals emanating from tumors can reprogram cancer-associated fibroblasts (CAFs), immune cells and endothelial cells, and promote extracellular matrix remodeling processes that lead to a favorable microenvironment for invasion, immune evasion, and therapy resistance. Altered expression of miRNAs in stromal fibroblasts has been demonstrated to promote the transformation of normal fibroblasts into CAF-like cells in ovarian cancer with alterations in miR-31, miR-214, and miR-155 associated with chemokine expression and stromal activation. Recent studies also support the concept that paracrine signals derived from ovarian cancer can convert normal fibroblasts into an activated CAF phenotype, characterized by enhanced secretory activity, increased contractility, remodeling of the extracellular matrix, and drug resistance-promoting functions. Besides fibroblast activation, hypoxia and metabolic reprogramming may further connect the tumor microenvironment to chromatin regulation. Although much of the direct evidence comes from other solid tumors, hypoxia-induced epigenetic alterations in stromal fibroblasts can drive glycolytic metabolic pathways, lactate synthesis, and tumor-promoting characteristics. These observations are relevant to ovarian cancer as hypoxic peritoneal and omental metastatic niches provide selective pressure for both malignant and stromal adaptation. Collectively, these data suggest that epigenetic therapy should be considered not only as an intervention targeting tumor cells but also as a possible approach to reconfigure tumor–stroma interactions, immune suppression, and metabolic support in the metastatic microenvironment [33–38].

Besides stromal remodeling mediated by fibroblasts, there is growing evidence that metabolic adaptation in the tumor microenvironment can directly affect on chromatin regulation and immune suppression. Examples of such metabolites include acetyl-CoA and α-ketoglutarate, which serve as essential cofactors for histone acetyltransferases and DNA/histone demethylases, thereby linking cellular metabolism to the regulation of epigenetic states. Hypoxia-induced metabolic reprogramming can foster the establishment of immunosuppressive chromatin landscapes in tumor-associated myeloid cells, resulting in reduced antigen presentation, T-cell dysfunction, and resistance to immunotherapy. These findings support an evolving model in which metabolic and epigenetic networks cooperate to drive ovarian cancer progression and therapeutic response [34,35].

DNA methylation also suppresses endogenous retroviral elements and double-stranded RNA sensing pathways to produce an immune-silent tumor phenotype. Chiappinelli et al. [39] showed that pharmacological DNA demethylation induces viral mimicry through reactivation of endogenous retroelements that stimulate interferon signaling and antigen presentation. This epigenetic-driven immune activation provides a mechanistic basis to introduce epigenetic therapies as immune sensitizers and not only as cytotoxic therapies. These therapies will be discussed in the following sections.

### DNA methyltransferase inhibitors (DNMTis)

Agents such as 5-azacytidine inhibit DNMT activity and reduce DNA methylation, and have been shown to be effective in re-sensitizing ovarian cancer cells to platinum-based therapies through re-activation of genes such as *BRCA1* and *MLH1* [2,13]. These compounds are especially important in tumors where DNA repair mechanisms are silenced by hypermethylation, causing resistance. DNMT inhibitors have been developed first for hematologic malignancies and are in the pipeline for solid tumors, often in combination approaches. Numerous studies indicate that demethylating agents are most effective when administered prior to or concomitantly with chemotherapy to induce epigenetic reprogramming and increase drug sensitivity [1,7].

### Histone deacetylase and methyltransferase inhibitors

Histone deacetylase inhibitors (HDACi) reactivate silenced genes involved in apoptosis and DNA damage response by restoring acetylation marks and promoting chromatin relaxation. HDAC inhibitors such as Vorinostat, Belinostat, and Panobinostat have shown promising effects in preclinical ovarian cancer models, particularly in reversing platinum-based therapies resistance [2,21]. Targeting of histone methyltransferases, in particular EZH2 that catalyses the repressive H3K27me3 mark, has also emerged as a viable strategy. EZH2 inhibitors have been shown to be effective in reducing tumor growth and re-sensitizing resistant cells to chemotherapy via de-repression of silenced tumor suppressor genes [9,19]. Novel epigenetic therapies under exploration include acetylated histone readers such as Bromodomain and extra-terminal motif (BET) inhibitors, and sirtuin modulators [8,16].

### MicroRNA-based therapeutics

MiRNA mimics and antagomirs (anti-miRNA oligonucleotides) are designed to restore or suppress specific miRNA activities. For example, restoration of tumor-suppressive miRNAs such as the miR-200 family or inhibition of oncogenic miRNAs such as miR-21 may overcome chemoresistance and reduce metastatic behavior [11,22]. The clinical translation of miRNA-based therapies still faces challenges in terms of delivery, stability, off-target effects, and immune activation, albeit promising. Research is ongoing on nanoparticle-based delivery systems and combination strategies to improve the therapeutic efficacy [4,12].

### Combination therapies and clinical trials

Combination therapies targeting different epigenetic pathways such as DNMT inhibitors with HDAC inhibitors or EZH2 inhibitors in combination with chemotherapy are being proposed to increase efficacy and reduce compensatory resistance mechanisms [1,3]. Many epigenetic agents are in early phase clinical trials for ovarian and other solid neoplasms. However, clinical application of these agents is limited by issues such as tumor heterogeneity, the lack of appropriate epigenetic biomarkers for patient stratification, and dose-limiting toxicities [8,15].

In solid tumors, the epigenetic reprogramming that initiates viral mimicry is a biological event that is conserved and replicable. The DNA demethylating agents activate normally silenced repetitive elements leading to innate immune signaling and prolonged interferon responses, as independently validated by Roulois et al. [40]. In the context of ovarian cancer, epigenetic immune priming may restore tumor immunogenicity and improve sensitivity to immune checkpoint blockade. Collectively, these observations support a translational framework where epigenetic therapies act upstream of immunotherapy, remodeling the tumor-immune interface to overcome intrinsic resistance [39,40].

Despite these promising mechanistic and preliminary clinical insights, there are several important limitations to the clinical application of epigenetic therapies in ovarian cancer. In the past, DNMT and HDAC inhibitors have been more effective in treating blood cancers than solid tumors. In solid tumors, these drugs have not been very effective on their own and have rarely led to long-lasting responses. These differences likely reflect a multitude of factors, including poor tumor penetration, dose-limiting hematologic and nonhematologic toxicities, lack of tumor-specific epigenetic selectivity, and the difficulty of achieving sustained pharmacodynamic modulation in the heterogeneous microenvironment of different solid tumors. Genome-wide epigenetic reprogramming may also affect non-malignant tissues, leading to concerns regarding toxicity to normal tissues and unintended destabilization of chromatin. These constraints reinforce the notion that epigenetic agents are more likely to be of clinical utility as biological priming agents or chemosensitizers than as universally effective monotherapies. A major hurdle is that changes in DNA demethylation and histone acetylation may be transient. Re-methylation and adaptive reconfiguration of chromatin may occur after cessation of treatment, and this may allow tumor cells to re-establish resistant transcriptional programs. Furthermore, the development of validated predictive biomarkers for most epigenetic agents is suboptimal, which hinders the identification of patients most likely to benefit. Future clinical development should focus on biomarker-enriched trials, pharmacodynamic end points, optimal biological dosing, rather than maximum tolerated dosing, and rational combinations with chemotherapy, PARP inhibitors, or immune checkpoint blockade [8,15,41–43].

## Clinical translation of epigenetic insights in ovarian cancer: trials, therapeutic strategies, and patient stratification

**Figure 2:**
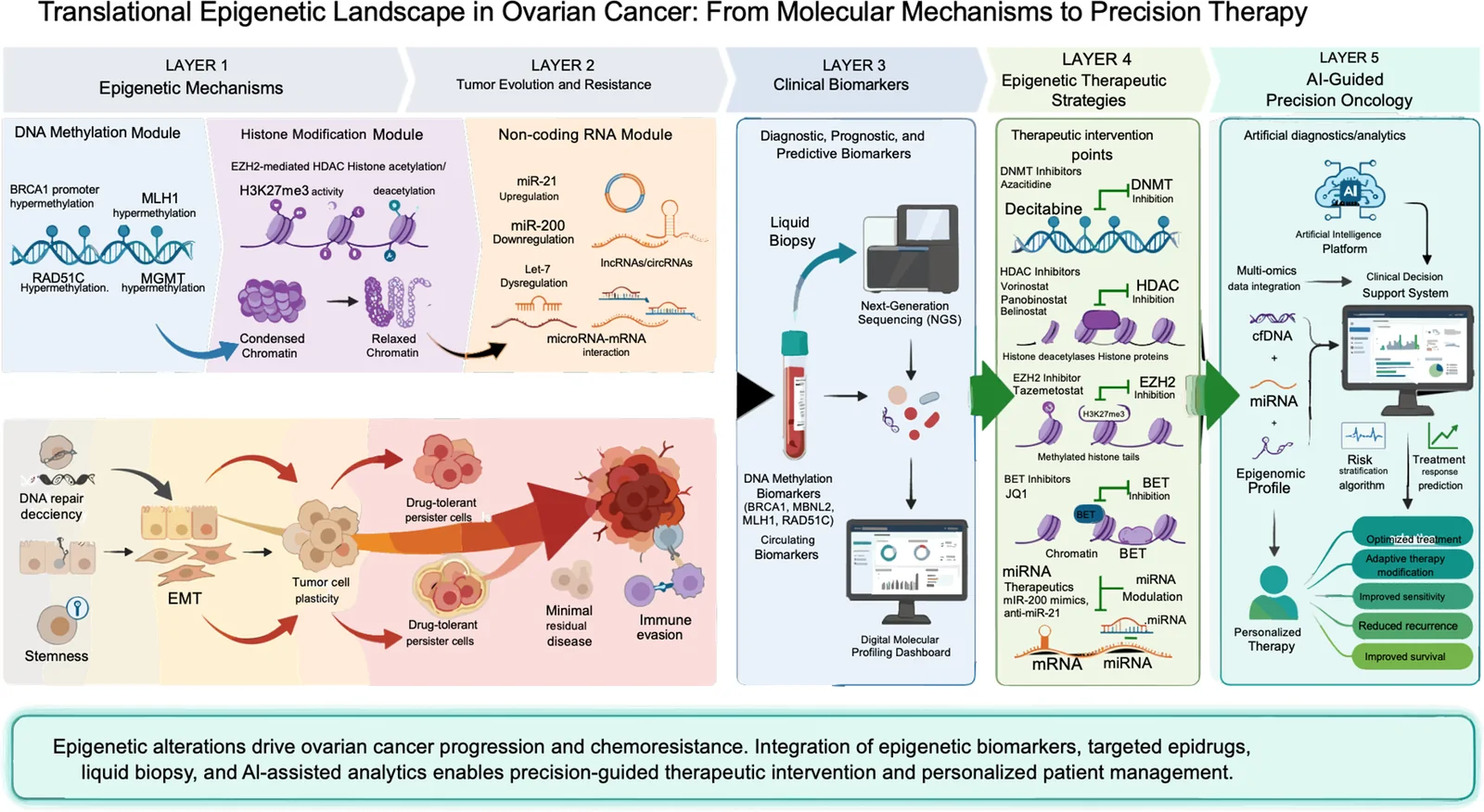
Translational epigenetic landscape in ovarian cancer: from molecular mechanisms to AI-guided precision therapy Figure 2 summarizes the progressive translation of epigenetic regulation into clinical application across five layers. Layer 1 illustrates core molecular mechanisms, including DNA methylation, histone modification, and miRNA dysregulation (e.g., BRCA1, MLH1, and miR-21), which drive transcriptional silencing and initiate tumor transformation. Layer 2 depicts disease progression, where epigenetically driven EMT and activation of drug efflux pumps promote chemoresistance and invasion. Layer 3 shows circulating biomarkers, methylated cfDNA and exosomal miRNAs which are detected through liquid biopsy for non-invasive diagnosis and therapy monitoring. Layer 4 highlights epigenetic therapies: DNMT inhibitors demethylate silenced genes, HDAC inhibitors restore histone acetylation, EZH2 inhibitors block H3K27me3 repression, and BET inhibitors suppress MYC driven transcription. Layer 5 represents AI-guided clinical translation, where biomarker data integrate into digital dashboards for personalized treatment prediction and adjustment. Together, the layers depict the translational continuum from molecular epigenetic alterations to precision-guided oncology, linking fundamental mechanisms with individualized ovarian cancer management.

The last decade has witnessed considerable change in ovarian cancer research from pure mechanistic epigenetics to translational and clinical applications. The advent of epigenetic pharmaceuticals (epidrugs) targeting DNMTs, HDACs, and EZH2 offers new avenues for overcoming chemoresistance and tailoring precision therapy. Epigenetic alterations are reversible and can be pharmacologically reprogrammed to restore drug sensitivity and immune recognition in tumor cells, unlike fixed genetic mutations [8,43,44]. Such insights have led to several clinical trials adding epidrugs to conventional platinum-based chemotherapy, PARP inhibitors, and immunotherapy regimens. We compare the major epigenetic therapeutic approaches, clinical trial outcomes, biomarker associations, and translational limitations in Table 5. This complete picture, illustrating the translational pathway from epigenetic mechanisms to clinical application in ovarian cancer, is outlined in (Figure 2).

**Table 5:** Clinical trials and translational context of epigenetic agents in ovarian cancer

Agent/Strategy	Target	Trial phase/Model	Population	Key outcome	Biomarker association	Major limitation/Toxicity	Translational interpretation
Azacitidine + carboplatin	DNMT/DNA methylation	Phase Ib–IIa	Platinum-resistant/ refractory EOC	ORR 13.8%; median PFS 3.7 months	Not clearly biomarker-enriched	Hematologic toxicity; small cohort	Suggests chemosensitization signal but not definitive efficacy
Decitabine priming + carboplatin	DNMT/DNA methylation	Phase II	Recurrent ovarian cancer	Response rate 35%; PFS 10.2 months	Demethylation of MLH1, BRCA1, HOXA11	Limited cohort; needs validation	Supports pharmacodynamic rationale for platinum resensitization
Guadecitabine-based strategies	DNMT/DNA methylation	Early phase/investigational	Recurrent/platinum-resistant disease	Partial reversal of resistance reported	DNA repair gene reactivation	Requires larger controlled trials	Improved pharmacokinetics but clinical role remains investigational
HDAC inhibitors + chemotherapy	HDAC/histone acetylation	Preclinical/early phase	Platinum-resistant models/patients	Reactivation of pro-apoptotic pathways	Limited predictive markers	Toxicity; modest single-agent efficacy	More plausible as combination/priming therapy
EZH2 inhibitors	EZH2/H3K27me3	Preclinical/early clinical	ARID1A-mutated clear-cell/endometrioid subtypes	Tumor suppression/disease stabilization	ARID1A mutation as candidate stratifier	Need subtype-specific clinical validation	Strong precision-therapy rationale
BET inhibitors + PARP inhibitors	BET proteins/acetyl-histone readers	Preclinical	Epithelial ovarian cancer models	Synergy with PARP inhibition	MYC/DNA damage response pathways	Preclinical evidence only	Promising but not yet clinically established

Table contents were compiled and interpreted from references cited within the original manuscript [1,8,21,37,41,42,44],

### Clinical trials of DNMT, HDAC, and EZH2 inhibitors

#### DNMT inhibitors

DNMT inhibitors such as Azacitidine, Decitabine, and Guadecitabine (SGI-110) have shown measurable but inconsistent efficacy in platinum-resistant ovarian cancer in clinical use. In a Phase Ib–IIa study, Fu et al. [45], reported that the combination of azacitidine and carboplatin resulted in an overall response rate of 13.8% and a median progression-free survival (PFS) of 3.7 months with manageable hematologic toxicity. Pre-treatment with Decitabine followed by carboplatin resulted in a 35% response rate and 10.2 months PFS in recurrent disease [44] Notably, these responses were associated with demethylation of *MLH1*, *BRCA1*, and *HOXA11* promoters, thus providing a mechanistic rationale for the use of DNA hypomethylation for chemo-sensitization. Pharmacokinetics and stability were further improved using Guadecitabine (SGI-110). The NCT02900560 trial demonstrated reactivation of DNA-repair genes and a partial reversal of platinum drugs resistance, confirming epigenetic resensitization without dose-limiting toxicities [36,46].

#### HDAC inhibitors

HDAC inhibitors such as Vorinostat, Belinostat, and Panobinostat have been evaluated as monotherapy and in combination regimens. Inhibition of HDACs reactivated pro-apoptotic genes and increased platinum-based therapies sensitivity in preclinical and Phase I studies [2,21]. HDAC inhibition alone showed limited clinical benefit but synergistically promoted chromatin relaxation and transcriptional reactivation when combined with DNMT inhibitors. Stone et al. [38] reported that combination of Azacitidine, an HDAC inhibitor and immune checkpoint blockade enhanced the interferon signaling and improved the survival in murine ovarian models. Analogous triplet regimens that combine epigenetic and immunotherapeutic agents are being tested in ongoing human trials [1].

#### EZH2 and BET inhibitors

The synthetic lethality between EZH2 inhibition and *ARID1A* mutations, has sparked interest in clear-cell and endometrioid ovarian cancers. Alldredge & Eskander [47] and Fan et al. [48] reported that EZH2 inhibitors (Tazemetostat, GSK126) suppress tumor growth and metastasis in these molecularly defined subtypes. An initial clinical evaluation (NCT01897571) confirmed that EZH2 inhibition is well tolerated and leads to disease stabilization in heavily pretreated patients [8].

Bromodomain and extra-terminal domain (BET) proteins (BRD2, BRD3, and BRD4) are epigenetic “readers” that recognize acetylated lysine residues on histone tails and drive transcriptional activation of oncogenic programs such as MYC-driven gene expression. Pharmacological BET inhibitors, such as JQ1 and OTX015 (MK-8628), disrupt interactions between chromatin proteins and possess antitumor activity in a range of solid tumors [49]. Preclinical studies of ovarian cancer have shown that inhibition of BET reduces proliferation and transcriptional programs associated with c-MYC, resulting in tumor cell death [50]. Also, BET inhibitors such as JQ1 have demonstrated synergistic activities with PARP inhibitors (e.g., Olaparib) in EOC models by promoting DNA damage and checkpoint [51].

Analysis of these clinical data must be performed with caution. Many trials of epidrugs in ovarian cancer have been early-phase and limited by small cohorts, single-arm designs, or lack of biomarker selection, which limits statistical power and makes cross-trial comparisons difficult. Therefore, reported response rates and PFS values should be considered as indicators of biological activity, not as conclusive proof of clinical efficacy in setting standards. Future trials should consistently report phase, sample size, composition of control arm, strategy of biomarker enrichment, pharmacodynamic evidence of target engagement, and toxicity profiles. Such contextualization is important in distinguishing between true therapeutic benefits and exploratory signals generated in highly selective, or underpowered, clinical settings [1,43–46].

### Epigenetic therapy in combination and resistance-reversal strategies

The combination of DNMT inhibitors and PARP inhibitors (e.g., Azacitidine + Olaparib) led to a median PFS of 19.1 months versus 5.5 months in the control arm, which highlights the potential synergetic effect via enhanced HRD as emphasized by Moufarrij et al. [43] and Wang et al. [1]. Trials in parallel with DNMT and HDAC inhibitors showed increased ability to reactivate transcription of tumor-suppressor networks, while EZH2 inhibition restored antigen presentation and allowed T-cell infiltration [48]. In addition, epigenetic agents can re-sensitize immune resistant tumors. Demethylating therapy up-regulates the expression of MHC class I, interferon-stimulated genes and *PD-L1*, making tumors more amenable to checkpoint blockade therapy [37]. This epigenetic immune convergence is a critical translational axis that could translate immunologically “silent” ovarian cancers into responsive phenotypes.

### Clinical validation of epigenetic biomarkers

#### DNA methylation panels

Robust methylation-based biomarkers are on the way to clinical application. Marinelli et al. [28] validated an 11-gene plasma methylation panel with 79% sensitivity and 96% specificity for detection of EOC. A CpG-rich panel (COL23A1, C2CD4D, WNT6) was shown by Widschwendter et al. [52] to distinguish high-grade serous ovarian carcinoma (HGSC) from controls with 90.7% sensitivity, while an EpiClass methylation-density classifier based on ZNF154 cfDNA methylation obtained 91.7% sensitivity and 100% specificity in an independent validation cohort [53]. These assays support the feasibility of methylation testing in liquid biopsies. Presently, prospective trials (e.g., NCT05420123) are evaluating cfDNA methylation as a companion diagnostic to predict responses to PARP and DNMT inhibitors.

#### Circulating and exosomal microRNAs

MicroRNA profiling improves DNA methylation diagnostics. In numerous validation studies it has been shown that circulating miR-21, miR-16-5p, and members of the miR-200 family have diagnostic and prognostic value. Timofeeva et al. [54] showed that miR-16-5p levels are predictive of surgical and chemotherapeutic outcomes with a specificity up to 90.9%. Ravegnini et al. [55] validated that circulating miR-135a-3p levels were associated with therapeutic response and PFS. Furthermore, Shiao et al. [56] reported that the combination of exosomal miR-1290 and CA-125 achieved AUC values close to 0.97, which was one of the most accurate non-invasive biomarkers so far. The integration of these biomarkers in clinical practice has the potential to revolutionize early diagnosis, risk assessment, and treatment monitoring.

#### AI-enhanced cfDNA and multi-omics platforms

The advent of machine learning has transformed biomarker interpretation. Bahado-Singh et al. [27] used artificial intelligence-based cfDNA methylation analysis and achieved 100% sensitivity and 72–100% specificity in the detection of ovarian cancer. Comparable AI-integrated epigenomic pipelines are being generated to combine cfDNA, miRNA, and lncRNA profiles to generate composite signatures with predictive accuracy >0.95 AUC [57,58]. Multimodal platforms allow the real-time monitoring of patients, possibly guiding therapy modifications when epigenetic reprogramming points to emerging resistance. The results are promising but caution should be exercised in the interpretation of AI-generated diagnostic metrics. Elevated sensitivity, specificity, or AUC values may be affected by cohort size, case–control design, feature selection bias, overfitting, and lack of independent external validation. Epigenomic classifiers combined with AI for clinical translation should be prospectively evaluated in diverse populations, compared with established markers such as CA-125 and HE4, and evaluated for calibration, reproducibility, interpretability, and clinical utility. Without such validation, these platforms should be regarded as emerging decision support tools and not as replacements for existing diagnostic and monitoring approaches [27,57,58].

### Clinical outlook and section summary

Early phase studies generate limited clinical data, but their impact on translation is substantial. Epigenetic therapy has demonstrated that the resistance phenotype of ovarian cancer can be reprogrammed. Future directions will rely on biomarker-driven patient selection, rational combination therapies, and integrated digital platforms to personalize epigenetic therapies. As phase II and III trials progress, the combination of DNMT, HDAC, and EZH2 inhibitors with immunotherapy or PARP inhibitors is expected to establish a new standard in recurrent or chemo-resistant diseases. The addition of liquid biopsy methylation panels and miRNA classifiers as predictive tools will enhance epigenetic precision oncology in routine gynecologic cancer management.

Identifying patients who are most likely to benefit from epidrug regimens remains a major clinical challenge. Molecular subtyping including *BRCA1* methylation, *EZH2* expression and miRNA signatures is used for trial participant selection Integrated multi-omics and AI modeling may be used by future algorithms to identify the most effective drug or combination therapy to assign patients to epigenetic responder phenotypes. The use of adaptive clinical designs in translational pipelines, in which sequential cfDNA methylation and miRNA profiling are used to inform treatment switches dynamically [1,27], is gaining momentum. The incorporation of validated epigenetic biomarkers into routine clinical practice may ultimately enable a paradigm shift in response-adaptive oncology from static to epigenetically-guided cancer therapy.

The clinical significance of epigenetic therapy in ovarian cancer is exploiting context-specific chromatin dependencies and immune modulatory effects. The loss of *ARID1A* results in vulnerabilities that can be exploited therapeutically because of epigenetic dysregulation, not genomic instability, and DNA methylation affects immune visibility and therapeutics. Collectively, these mechanisms support stratifying patients with epigenetic states, including chromatin remodeling deficiencies and immune epigenetic signatures, to guide the administration of epidrugs in a systematic manner. This strategy is also in line with the concepts of precision oncology, targeting reversible regulatory processes rather than stable genetic alterations [20,39].

Many practical challenges need to be overcome before epigenetic precision oncology can be incorporated into routine treatment of ovarian cancer. Methylation companion diagnostics require stringent analytical standardization, such as standardized pre-analytical plasma processing, cfDNA isolation, bisulfite conversion or sequencing protocols, assay cut-offs, and inter-laboratory reproducibility. Differences in tumor fraction, sample timing, treatment exposure, and biological compartment can greatly affect cfDNA methylation and circulating RNA measurements. Second, for the most part, epigenetic biomarkers have not been validated sufficiently as predictive tools for the selection of epidrugs and are often used more as exploratory or prognostic tools rather than as clinically approved companion diagnostics. Integration of regulatory and clinical pathways remains a challenge. Epigenetic assays must show clinical validity, clinical utility, and cost-effectiveness before being introduced into guideline-conformant protocols for ovarian cancer. This is particularly important as current management is driven by well-established tools including histology, staging, CA-125 kinetics, *BRCA1/2* mutation status, HRD testing, platinum-free interval, and eligibility for PARP inhibitors. Future implementation will require prospective validation of epigenetic biomarkers, definitive decision thresholds, standardized reporting formats, and demonstration that epigenetically guided treatment modifications improve outcomes beyond current clinical and molecular stratification methods [27,28,41,42,52,53,57,58].

The limited translation of epigenetic therapies from early phase trials to routine clinical application is likely a consequence of the biological complexity of solid tumors rather than a failure of epigenetic targeting. In ovarian cancer, tumor cells may evade long-term therapeutic suppression via a diversity of chromatin states, restricted target engagement, adaptive transcriptional reconfiguration, and rapid epigenetic reprogramming, even when initial responses are seen. In addition, non-specific DNMT and HDAC inhibitors often display a lack of tumor selectivity, increasing the risk of systemic toxicity, and limiting the ability to achieve sustained biologically effective dosing. These observations suggest that future epigenetic therapies will likely require biomarker-guided patient selection, combinatorial strategies, and dynamic longitudinal monitoring rather than empirical monotherapy approaches [41,42].

## Future directions in personalized epigenetic therapy

The continuous evolution of oncology toward precision medicine holds great promise for the incorporation of epigenetic data in personalized treatment strategies for ovarian cancer. Defining patient-specific epigenomic profiles allows for the development of tailored interventions, risk stratification tools, and predictive biomarkers specific to the individual biology of the tumor [1,3]. Although there has been promising progress, the use of personalized epigenetic therapy in the clinic has many technical, biological, and translational challenges.

### Comprehensive epigenomic profiling

The whole genome mapping of DNA methylation, histone modifications and ncRNA expression in ovarian tumors were made possible by high-throughput technologies such as whole genome bisulfite sequencing, ChIP-seq, and RNA-seq. Such profiles can reveal unique epigenetic subtypes, identify actionable modifications (e.g., *BRCA1* hypermethylation, *EZH2* overexpression), and guide the choice of epigenetic therapeutics or combinations with chemotherapy [2,5].

Future strategies may combine epigenetic signatures into molecular classification systems for improved prediction of therapeutic response and long-term prognosis (Table 6). For example, detection of tumors with specific signatures of *MBNL2* hypomethylation or increased circulating miR-21 levels could be used for early intervention or combination therapy strategies [18,22].

**Table 6:** Future directions in personalized epigenetic therapy

Innovation area	Strategy	Role in personalization	Challenges
Epigenetic editing	CRISPR/dCas9-DNMT3A/TET1	Locus-specific gene reprogramming	Delivery, specificity
Multi-Omics integration	AI/ML modeling of methylation, miRNA, histone data	Therapy prediction & stratification	Data standardization
Liquid biopsies	Circulating miRNAs, methylated cfDNA	Non-invasive monitoring	Sensitivity/specificity issues

### Epigenetic editing and CRISPR-based technologies

CRISPR/dCas9-mediated epigenome editing offers an exciting experimental platform for locus-specific regulation of gene expression without changing the underlying DNA sequence. In theory, Cas9 that is catalytically inactive and fused to epigenetic effectors such as DNMT, TET, histone acetyltransferase, or histone methyltransferase domains could silence oncogenic loci or reactivate tumor suppressor genes that are epigenetically silenced. However, this methodology is still largely preclinical in ovarian cancer and needs to be distinguished from clinically applicable epigenetic therapy. Key translational hurdles involve effective delivery to widespread peritoneal and metastatic tumor sites, specificity to target cells, stability of edited chromatin states, potential off-target chromatin effects, immunogenicity of delivery vehicles, and regulatory review of long-term safety. Therefore, CRISPR/dCas9 epigenome editing should be currently regarded as a mechanistic and investigational platform rather than a direct therapeutic approach [59–61].

In addition to technical limitations, clinical application of epigenome editing strategies also requires careful ethical and regulatory considerations, in particular with regard to long-term chromatin stability, unintended heritable transcriptional effects and persistent off-target epigenetic modifications. The regulatory pathways for programmable epigenetic editing are not well defined, especially for therapies seeking to induce persistent chromatin state changes in heterogeneous solid tumors [59–61].

### Artificial intelligence and multi-omics integration

Understanding complex epigenetic data relies more and more on artificial intelligence (AI) and machine learning. Integrative models of epigenomic, transcriptomic, proteomic, and clinical data can enhance the prediction of therapeutic response, recurrence risk, and patient stratification [1,26]. Integration of multiple omics allows identification of novel therapeutic vulnerabilities and drug combination strategies. Real-time monitoring of epigenetic alterations by AI-assisted modeling may soon be possible to adapt therapy to developing resistance mechanisms.

### Clinical translation and future trials

Future clinical trials must incorporate biomarker-driven designs to fully realize the potential of personalized epigenetic therapy. Patient selection should be based on epigenetic alterations (e.g. *BRCA1* methylation, *EZH2* expression, and miR-200 status) to maximize therapeutic efficacy and minimize unnecessary toxicity [8,15]. An emerging frontier is the exploration of epigenetic agents in combination with immunotherapy, PARP inhibitors, and targeted therapies. Addressing current challenges including tumor heterogeneity, delivery issues, and lack of validated companion diagnostics will be critical for incorporation of these therapies into standard clinical practice.

## Conclusion

Epigenetic regulation is central to the development, progression and therapeutic resistance of ovarian cancer. Previously, these reversible changes were assumed to be purely mechanistic, but now there is new clinical evidence that they are amenable to interventions. DNA methylation and histone modification have transitioned from molecular features to predictive biomarkers that can inform therapeutic decisions. Translational markers such as methylation of *BRCA1* and *MLH1* promoters, EZH2-mediated H3K27me3 and circulating miRNAs including the miR-21 and miR-200 families serve as promising tools to guide prognosis, therapy selection and response monitoring.

The clinical use of epigenetic drugs has passed the proof-of-concept stage. DNMT, HDAC, and EZH2 inhibitors alone or in combination with PARP and immune-checkpoint inhibitors are showing the potential to resensitize resistant tumors and increase immunogenicity. At the same time, cfDNA- and exosome-based tests with AI-augmented analytics allow for dynamic, non-invasive monitoring of treatment effectiveness.

The future of ovarian cancer management is in biomarker-driven adaptive therapy frameworks integrating mechanistic understanding and clinical application. This vision requires rigorous validation of epigenetic signatures, systematic trials of drug combinations, and thorough integration of multi-omics and digital health technologies. The combination of lab epigenetics and patient-specific precision oncology is a rational evolution of the discipline and a plausible opportunity to counteract chemoresistance and improve survival in ovarian cancer. The identification of epigenetically driven tumor plasticity and MRD underscores the possibility of reversible chromatin states that may favor relapse and therapeutic escape, even after initial treatment response.

## Artificial Intelligence (AI) Disclosure

Figure Labs AI was used solely to assist in the graphical design and layout of figures in this manuscript. The authors provided the scientific concepts, figure content, and figure structure, and subsequently reviewed, edited, and validated all graphical elements for accuracy and consistency with the cited literature.

## Abbreviations

BET: bromodomain and extra-terminal motif
CAF: cancer-associated fibroblast
ceRNA: competing endogenous RNA
cfDNA: cell free DNA
circRNA: circular RNA
DNMT: DNA methyltransferase
DNMTi: DNA methyltransferase inhibitor
EMT: epithelial–mesenchymal transition
EOC: epithelial ovarian cancer
EZH2: enhancer of zeste homolog 2
HGSC: high-grade serous ovarian carcinoma
HDAC: histone deacetylase
HDACi: histone deacetylase inhibitor
HRD: homologous recombination deficiency
HRR: homologous recombination repair
lncRNA: long non-coding RNA
miRNA: microRNA
MRD: minimal residual disease
ncRNA: non-coding RNA
PARP: poly(ADP-ribose) polymerase
PFS: progression-free survival
TET: ten-eleven translocation
